# Identification of transmission chains and clusters associated with COVID-19 in Tunisia

**DOI:** 10.1186/s12879-021-06107-6

**Published:** 2021-05-19

**Authors:** Mouna Safer, Hejer Letaief, Aicha Hechaichi, Chahida Harizi, Sonia Dhaouadi, Leila Bouabid, Sondes Darouiche, Donia Gharbi, Nawel Elmili, Hamida Ben Salah, Mongi Hammami, Khouloud Talmoudi, Rim Moussa, Nejib Charaa, Hasna Termiz, Fethi Ltaief, Habib Tounekti, Mohamed Makhlouf, Asma Belguith Sriha, Manel Ben Fredj, Sonia Khalfallah, Houcine Jabrane, Selma Mchirgui, Chedli Amich, Radhia Dabghi, Zid Anez, Latifa Abdelkader, Moncef Mhamdi, Nabil Ouerfeli, Salah Zoghlami, Souha Bougatef, Mohamed Kouni Chahed, Nissaf Bouafif Ben Alaya

**Affiliations:** 1 National Observatory of New and Emerging Diseases, Ministry Of Health, Tunis, Tunisia; 2 Cardio Vascular Disease Epidemiology and Prevention Research Laboratory, Faculty of Medicine, University of Tunis El Manar, Tunis, Tunisia; 3 Department of Epidemiology and Statistics, Abderrahman Mami Hospital, Ariana, Tunisia; 4 Public Health Surveillance Regional Departments, Tunis, Tunisia; 5 Department of Community and Preventive Medicine Faculty of Medicine, University of Monastir Tunisia, Tunis, Tunisia; 6 Department of Epidemiology and Public Health, Faculty of Medicine of Tunis, Tunis El-Manar University, Tunis, Tunisia

**Keywords:** COVID-19, Coronavirus infections / transmission, Contact tracing, Cluster analysis, Public health, Tunisia

## Abstract

**Background:**

The aim of this study was to characterize the transmission chains and clusters of COVID-19 infection in Tunisia.

**Methods:**

All cases were confirmed by Reverse Transcriptase Polymerase Chain Reaction of a nasopharyngeal specimen. Contact tracing is undertaken for all confirmed cases in order to identify close contacts that will be systematically screened and quarantined. Transmission chains were identified based on field investigation, contact tracing, results of screening tests and by assessing all probable mode of transmission and interactions.

**Results:**

As of May 18, 2020, 656 cases out of a total of 1043 confirmed cases of Coronavirus disease 2019 belong to 127 transmission chains identified during the epidemic (mean age 42.36 years, Standard deviation 19.56 and sex ratio 0.86). The virus transmission is the most concentrated in the governorate of Tunis (31.5%), Ariana (10.2%) and Ben Arous (10.2%). Virus transmission occurred 50 times (9.72% of secondary transmission events) between two different governorates. A maximum of seven generations of secondary infection was identified, whereas 62% of these secondary infections belong the first generation. A total of 11 “super spreader” cases were identified in this investigation. Four large clusters have been identified. The evolution of secondary cases highlighted two peaks: one in 2nd April and a second in 16 ^th^ April whereas imported cases caused local transmission of virus during the early phase of the epidemic.

**Conclusion:**

Correct contact tracing and early active case finding is useful to identify transmission chains and source of infection in order to contain the widespread transmission in the community.

## Background

Since December 2019, the world is experiencing a serious health crisis caused by the emergence of a new coronavirus, known SARS-CoV-2 as “Severe Acute Respiratory Syndrome 2” causing a respiratory distress syndrome, called COVID-19 as “Coronavirus disease 2019” [1]. Isolated for the first time in the city of Wuhan in the province of Hubei in China, this virus has already demonstrated its high infectivity even during the asymptomatic phase and its potential to generate explosive outbreaks [2, 3]. As of May 20, more than 300 countries and territories have reported cases of COVID-19, including more than 4 million confirmed cases and over 300,000 attributable deaths worldwide [4]. In response to this “alarming levels of spread and severity”, the World Health Organization (WHO) declared COVID-19 a public health emergency of international concern on 30 January 2020 [5] and then a pandemic on 11 March 2020 [6].

One of strategic objectives adopted by WHO in the response to COVID-19 rapid global spread was to “Interrupt human-to-human transmission including reducing secondary infections among close contacts and health care workers, preventing transmission amplification events, and preventing further international spread” [7] . The International Health Regulations (IHR) Emergency Committee stated in its second meeting that it “believes that it is still possible to interrupt virus spread, provided that countries put in place strong measures to detect disease early, isolate and treat cases, trace contacts” [8].

On March 2, 2020, Tunisia reported its first confirmed case of COVID-19 infection. in a 40-year-old man who had a history of travel in Italy [9]. Since this date, other imported cases were identified, followed by chains of secondary infection. As of May 18, 2020, the cumulative number of confirmed COVID-19 cases was 1043(252 imported and 791 local) corresponding to a cumulative incidence of 8.80/100000. Among COVID-19 confirmed cases 17.1% were still active. Fatality rate was 4.4% and the proportion of health care professionals infected was 13.6%.

Early preventive measures were put in place by the Tunisian government since 22 January 2020 (screening in points of entry and systematic 14 days isolation of travelers returning from risk areas). First preventive measures after the confirmation of the first case have been implemented (9–20 March 2020) including partial border closure, closure of schools and universities and curfew from 6 pm to 6 am(since 18 th March). On March 20, a national lockdown, was applied until 4 May the date of implementation of a targeted and progressive containment (return of commercial activities: 50% for large companies and 100% for small ones).

As it’s well documented that identification of the chain of disease transmission and the source of infection are crucial for guiding effectively containment measures [10], investigation was immediately activated to identify cases and contacts and to analyze transmission and super spreading events.

The aim of this study was to characterize the transmission chains and clusters of COVID-19 infection in the current Tunisian outbreak until May 2020.

## Methods

### Study design and population

This outbreak investigation was carried out as collaboration between the national level (Observatory of New and Emerging Diseases) and the regional level (Public Health Surveillance Departments). It concerned all patients with confirmed SARS-CoV-2 infection and close contacts of these patients.

### Definitions

MERS-CoV-2 infection was confirmed by positive real-time Reverse Transcriptase Polymerase Chain Reaction (RT-PCR) assays in one nasopharyngeal sample. Close contacts were defined as person having any contact within 1 m and cumulative face-to-face contact exceeding 15 min with a COVID-19 confirmed case. The day of symptom onset was defined as the day when any symptom (specific or nonspecific) occurred. A transmission pair was defined when two confirmed COVID-19 cases meet the following criteria:1/A clear epidemiologic link was established; 2/the infectee patient don’t have a history of travel to an area affected by COVID-19; 3/all other confirmed cases with an exposure history that didn’t allow to precise the source of the infection were excluded. So the transmission of an infectee patient can be only attributed to one infector whereas a patient could be infectee and infector in the same time.

Tracing and screening strategy: The contact tracing strategy was based on the rapid identification of all symptomatic and asymptomatic close contacts, application of self-isolation for 14 days and systematic screening of COVID-19 based on RT-PCR test. All close contacts in self-isolation were surveilled in order to detect symptoms indicating a second test. Quarantine was undertaken in home for close contacts and in specific structures for confirmed cases.

### Data sources and identification of transmission chains and infector/infectee pairs

Data allowing the analysis of transmission chains were the following:

1/ Individual-level data on infected patients was extracted from the “Tunisian daily COVID- 19 confirmed cases database”: the main following variables were extracted: age, sex, date of reporting case, date of symptom onset and the type of transmission of index cases (local or imported); 2/In Tunisia, as soon as a COVID-19 case was confirmed contact tracing was immediately undertaken by assessing activity patterns, from 14 days preceding symptom(s) onset until isolation and active case-finding among close contact was performed. All contacts were placed under quarantine for 14 days from last exposure to the individual with confirmed COVID-19 at home.

In a first stage the contact and exposure history was based on case interviews made by the regional investigator with the patients and/or their family members. As a second stage, in order to identify the final transmission chains and clusters and to precise different generations of secondary transmission, an epidemiologist of national level discussed with the field regional epidemiologist collaborator or directly with patients whose index cases or circumstances of transmission are uncertain.

### Identification of transmission chains and infectee / infector pairs

Activity maps collected in the contact tracing was analyzed and cross checked with data collected during additional investigation concerning circumstances and dates of exposure in order to identify links between cases and clusters.

Generations of secondary infection were defined as following: 1/Secondary cases were classified as first-generation infections if there was a history of direct contact with the index patient; 2/ for the generations following the first one, we defined “n” generation infection referring to those with exposure to “n-1” confirmed patients.

The infectee/ infected pairs were directly deduced from the transmission chains specifying all the generations of secondary transmission.

### Analysis of transmission dynamic

The main characteristic of transmission chains was summarized: total of transmission chains, total of secondary infections, generations of secondary infection, imported primary cases, infector/infectee pairs. The four largest clusters were analyzing by specifying the cluster location, the cluster size, the transmission mode of primary case, reporting date of first and last cases and events occurring the transmission in the cluster. We identified “super spreader” case of COVID-19 and we use for this the same definition adopted by the epidemiologists during the SARS outbreak: “super-spreader” is individual with transmission of SARS to at least eight contacts [11]. Drawing of transmission chains was performed using NetDraw 2.158 [12].

### Ethics approval

As conducted in response to a public health emergency, this epidemiological investigation, analysis of data and containment measures implemented in order to control the spread of the outbreak are exempted from ethical approval. Confidentiality of data was performed using key.

## Results

**Fig. 1:**
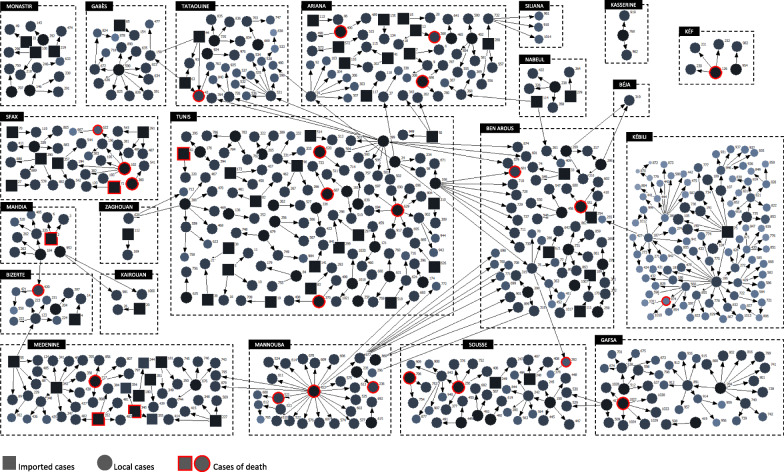
Cases were differentiated by respective generations of secondary infection (as of May 18 2020), origin of transmission (local/imported) and governorates. A total of 643 cases were presented after excluding 13 unclassified ones. Different generations were presented with circles of different size and a different shade of blue color (from the first to the seventh generation of secondary transmission the circles are smaller and less dark)

As of May 18, 2020, 656 cases (133 index cases and 523 cases of secondary infection) out of a total of 1043 confirmed cases of COVID-19 belong to 127 transmission chains identified during the epidemic (mean age 42.36 years, Standard deviation 19.56 and sex ratio 0.86). The analysis of transmission mode among primary cases showed a proportion of 44.1% of imported cases (Table 1, Fig. 1).

**Table 1:** Characteristic of 127 transmission chains of COVID 19, Tunisia March–May 2020

Governorate	Transmission chains		Imported primary cases		Secondary transmission cases (Maximum)	Generation of secondary transmission (Maximum)	Infector-infectee pairs	Infector and infectee COVID 19 case	Super spreader cases
	n	%	n	%					
**Tunis**	40	31.5	15	37.5	23	4	121	13	3
**Ariana**	13	10.2	9	69.2	6	2	28	2	–
**Ben arous**	13	10.2	4	30.8	5	2	33	3	–
**Medenine**	9	7.1	7	77.8	11	2	33	2	1
**Sfax**	9	7.1	6	66.7	9	3	24	3	–
**Sousse**	8	5.5	2	28.6	12	2	37	3	–
**Gafsa**	5	4.7	–	–	19	3	34	4	1
**Monastir**	6	4.7	3	50.0	4	2	10	1	–
**Tataouine**	4	3.1	1	25.0	5	2	19	2	–
**Bizerte**	3	2.4	2	66.7	10	2	11	0	–
**Mahdia**	3	2.4	1	33.3	8	3	9	1	–
**Manouba**	3	2.4	–	–	32	3	33	0	1
**Gabes**	2	1.6	1	50.0	12	3	14	3	1
**Kef**	2	1.6	–	–	3	1	4	0	–
**Nabeul**	3	1.6	1	50.0	3	2	4	0	–
**Zaghouan**	2	1.6	1	50.0	1	1	1	0	–
**Kairouan**	1	0.8	1	100.0	1	1	3	0	–
**Kasserine**	1	0.8	–	–	2	1	2	0	–
**Kebili**	1	0.8	1	100.0	106	7	93	29	4
**Total**	**127**	**100.0**	**55**	**43.3**	**106**	**7**	**513**	**66**	**11**

**Fig. 2:**
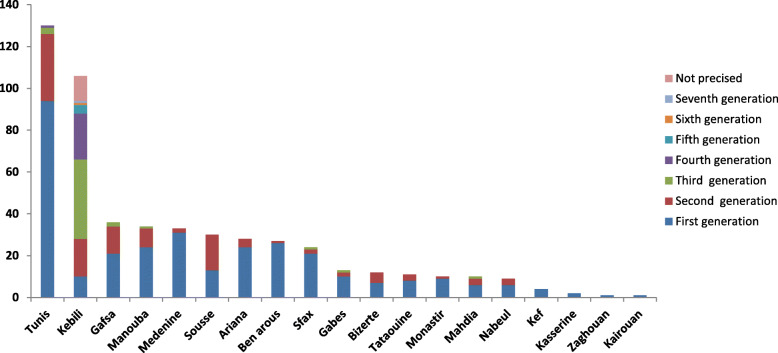
Generation of secondary infection of COVID-19 by governorate, Tunisia March–May 2020

**Fig. 3:**
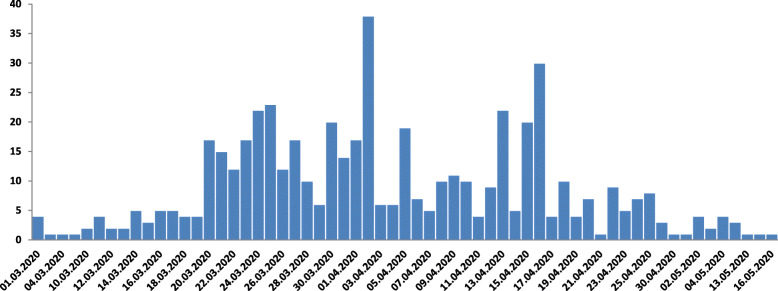
Confirmed cases of secondary infection among documented transmission chains of COVID 19, Tunisia March–May 2020

**Fig. 4:**
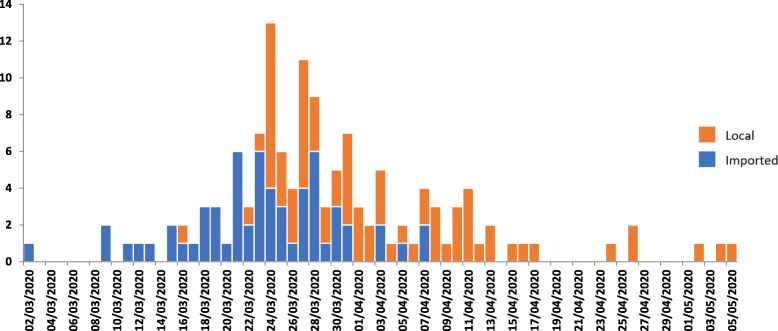
Confirmed index cases among documented transmission chains of COVID 19, Tunisia March–May 2020

The distribution of these chains by governorate was summarized in Table 1: the virus transmission is the most concentrated in the governorate of Tunis (31.5%), Ariana (10.2%) and Ben Arous (10.2%). Virus transmission occurred 50 times (9.72% of secondary transmission events) between two different governorates. The median of secondary cases linked to the index case was 2 (minimum of 1 and maximum of 106). A total of 513 infector-infectee pairs were identified with 66 COVID-19 positive cases at the same time infector and infectee. A maximum of seven generations of secondary infection was identified when establishing the transmission chains that took place in the governorate of Kebili whereas 62% of these secondary infections belong the first generation (Fig. 2). A total of 11 “super spreader” cases were identified in this investigation occurring 123 secondary transmission events (Table 1). Among these super spreaders ten were symptomatic the range of super spreader’s ages was 28–80. Four large clusters with a number of secondary cases exceeding 20 have been identified. The main characteristics, circumstances and dynamics of the transmission occurring in these clusters were shown in Table 2. The evolution of secondary cases during this investigation is represented in Fig. 3. This evolution highlighted two peaks: one in April 2 and a second in April 16. The evolution of index cases and their distribution in local/imported was shown in Fig. 4, imported cases caused local transmission of virus during early phase of the epidemic (until 20 March 2020 there were 67% of imported cases among the total index cases). A total of 28 deaths were reported among the 656 cases belonging transmission chains (Fig. 1).

**Table 2:** Characteristics of the largest COVID-19 clusters in Tunisia March–May 2020

Cluster identification	Cluster location	Cluster size	Transmission mode of primary case	Generation of secondary transmission	Reporting date for the first case	Reporting date for the last case	Transmission events
**Cluster 1**	Kebili	108	Imported	7	21/03/2020	05/05/2020	Family gathering/wedding ceremony
**Cluster 2**	Manouba	33	Local	3	31/03/2020	03/04/2020	Family gathering
**Cluster 3**	Tunis	24	Local	3	31/03/2020	24/04/2020	Family gathering
**Cluster 4**	Tunis	22	Local	3	16/03/2020	03/04/2020	Conferences/meetings

A total of ten healthcare associated COVID-19 infections were documented corresponding all to one generation of secondary infection. (Two transmissions from health professional to patient; 5 transmissions between health professional and 3 transmissions from patient to health professional).

## Discussion

In response to the rapid global spread of COVID-19 and in presence of local secondary infections, the understanding of the transmission dynamics of the virus is crucial to guide effectively containment measures and interrupt the spread of the virus. In this context, this is the first study documenting transmission chains and clusters of SARS-CoV-2 in Tunisia. This investigation showed how contact tracing and early investigation, upon a case of COVID-19 was confirmed, was crucial to document locally transmission and identify transmission chains. Using these methods, most possible sources of exposure could be identified and most clusters could be documented. This surveillance method is indicated to identify cases in the community and this will be missed if we focus only on symptomatic person and it is the most effective way to enable containment measures in order to stop super spreading events and transmission of the virus [10].

Analysis of transmission chains at early stage of the epidemic before implementation of control measures highlighted the sustained transmission of the SARS-CoV-2 in Tunisia. In fact, 47% of secondary cases were detected during the first month of the spread the virus in Tunisia (March). This sustained transmission was demonstrated also in a recent publication [13] which concluded to a temporal reproduction number R_0_ of 3.18, 95% CI [2.73–3.69].

The evolution of secondary cases showed two peaks: the first one occurring on April 2 was probably related to the lack of compliance to home quarantine among travelers coming from high-risk countries. Whereas the second peak, of April 16 could be the consequence of the delayed effect of non-compliance with general confinement measures related to mass gatherings as weddings, meetings and funerals.

The “Super spreaders” identified in this investigation have significantly participated in the spread of the disease. In fact, these 9 “Super spreaders” had transmitted the infection to a total of 95 close contact people. Similar events were associated with outbreaks of SARS and MERS [11, 14, 15] and reported recently with SARS-CoV-2 [16].

Concerning the evolution of the index cases, we have noted that imported cases contribute to secondary disease transmission in Tunisia at the early phase of the epidemic. This finding support the impact of government measures put in place such as travel restrictions since March 20 and mandatory quarantine of repatriated population since March 22.

As a consequence of the mounting number of COVD-19 cases in Tunisia and the sustained transmission, the government implemented many control measures from 18 March: curfew, lockdown, travel restrictions, social distancing measures including restriction of public transportation, cancelling of social and mass gathering events, school and university closure, and some professional activities as well as promotion of preventive measures among general population (barrier measures, physical distance, hand washing) and among health workers (Personal Protective Equipment and care hygiene).

In this study the four largest clusters showing a local transmission were analyzed. Transmission of infection in these clusters was probably attributed to close and prolonged contacts with symptomatic case and was occasioned by family gatherings (clusters 1, 2 and 3), a wedding ceremony (cluster1), a conference and meetings (cluster 4). Direct physical contact and handshaking was reported in family gatherings and meetings. In the wedding event (cluster 1), and one of family gatherings (cluster 1) sharing of meals during a ceremonial dinner was also reported.

These findings are similar to those shown in other published case series analyzing transmission chains. In fact, transmission by close contact is known as the most common transmission mode particularly when contact is prolonged [17]. Whereas the possibility of indirect transmission can’t be excluded since surface contamination and indirect transmission (via fomites and shared food) has been documented in some studies and this evidence emphasizes the importance to sensitize community to adopt strict personal and hand hygiene as a key preventive measure [18].

We recognized that this study had some limitations and some results should be interpreted carefully. First, as symptom onset dates and close contacts were self-reported, there could have been reporting information bias.

To mitigate these biases we collected information immediately after case confirmation. However, biases concerning the identification of close contacts could not be completely eliminated since this identification is mainly based on the statements of patients and contacts, nevertheless we explained the exact meaning of a “close contact” to all interviewees and we collected all the exposure history in order to define accurately all close contacts.

Second limitation concerned the use of only RT-PCR testing in active case-finding among close contacts. This method is known to offer a rapid diagnostic solution, but it can only detect SARS-CoV-2 during the period of viral shedding which still not certain and RT-PCR is so limited by its ability to detect convalescent cases of COVID-19 [19]. The first preliminary analysis [20] of SARS-CoV-2 IgM and IgG indicated that the antibody response in COVID-19 patients is similar to seroconversion kinetics indicated with Middle East respiratory syndrome (3 weeks after symptoms started) [21] and with severe acute respiratory syndrome (93% of patients seroconverted at an average of 20 days from symptom onset) [22], if not earlier than, these times.

Serological testing were demonstrated in a recent study [17], to have a crucial role in identifying convalescent cases or people with milder disease who might have been missed by RT-PCR and helped so to establish connections between COVID-19 clusters. This will be useful for epidemiological investigation by identifying more accurately infected people in clusters and track transmission dynamics which would better inform disease control policies for more or less containment efforts. It is also crucial to determine more accurately the number of infected patients because this will influence all other epidemiological estimations: such attack rates and case fatality rate.

Some difficulties were noted during this first step of outbreak investigation aimed to identify transmission chains of COVD-19. Difficulties concerned mainly the use of traditional epidemiologic methods to establish links between cases and clusters, which were sources of delays and inaccuracy to obtain information from cases and contacts so that the implementation of tracking device that will help to achieve contact tracing in a precise and valid manner is necessary in the next steps of the management of the epidemic.

The other perspectives in the next phases of epidemic management allowing to improve the identification of links between cases and between clusters concern adoption of additional laboratory techniques, such as serological tests and phylogenetic analysis, as well as the acquisition and activation of applications allowing accurate detection of close contacts and quarantine violators, enabling identification new close contacts in a timely manner.

## Conclusion

All the efforts made during the management of the epidemic of COVID-19 in Tunisia by conducting properly contact tracing and active case-finding facilitated the documentation of transmission chains and the identification of sources of infection. This rigorous approach was of great help to guide the containment measures and stop the spread of the epidemic and supports evidence that these measures were effective.

## Data Availability

The National Observatory of New and Emerging Diseases (Tunisia) as well as the corresponding author had full access to all data. This data wasn’t publicly accessible but available from the corresponding author upon reasonable request.

## Abbreviations

COVID-19: Coronavirus Disease 2019
RT-PCR: Reverse Transcriptase Polymerase Chain Reaction
SARS-CoV-2: Severe Acute Respiratory Syndrome Corona Virus 2
SARS: Severe Acute Respiratory Syndrome
IgM: Immunoglobulin M
IgG: Immunoglobulin G
